# 1,3,4-thiadiazole derivatives as PI3Ks inhibitor: design, in silico studies, synthesis, characterization, and antimicrobial evaluation

**DOI:** 10.55730/1300-0527.3732

**Published:** 2025-02-24

**Authors:** Dharmvir SINGH, Pankaj KUMAR, Anoop KUMAR, Vivek V. BHOSALE, Kalicharan SHARMA, Deepak KUMAR, Ramchander KHATRI, Tanuj HOODA, Amit LATHER

**Affiliations:** 1 Department of Pharmaceutical Chemistry, Ch. Devilal College of Pharmacy, Jagadhri, Haryana, India; 2 Department of Pharmaceutics, Dharamputra College of Pharmacy, Sonepat, India; 3 Department of Pharmacology, Delhi Pharmaceutical Sciences and Research University, New Delhi, Delhi, India; 4 Division of Toxicology and Experimental Medicine Division, CSIR-Central Drug Research Institute, Lucknow, India; 5 Department of Pharmaceutical Chemistry, ISF College of Pharmacy, Moga, Punjab, India; 6 Department of Pharmaceutical Chemistry, School of Pharmaceutical Sciences, Shoolini University, H.P, India; 7 Delhi Pharmaceutical Sciences and Research University, New Delhi, Delhi, India; 8 Department of Pharmaceutical Chemistry, MM College of Pharmacy, Maharishi Markandeshwar (deemed to be University), Mullana, India; 9 Department of Pharmacology, Geeta Institute of Pharmacy, Geeta University, Panipat, Haryana, India

**Keywords:** Docking studies, MMGBSA, PDB: 7JWE, 5-(pyridin-4-yl)-1, 3,4-thiadiazol-2-amine, in vitro antimicrobial activity

## Abstract

Since PI3Ks are targeted by a variety of bacterial pathogens, they represent a promising target for host-directed immune therapy and may be beneficial in managing persistent bacterial infections. In the present study, computational studies of 5-(pyridin-4-yl)-1,3,4-thiadiazol-2-amine derivatives for phosphoinositide-3-kinases (PI3Ks) inhibitors were carried out using dock scores, Glide scores, and the MMGBSA dG method, with comparison to standard drugs (ofloxacin and fluconazole). A series of 5-(pyridin-4-yl)-1,3,4-thiadiazol-2-amine derivatives (D1–D17) were synthesized and evaluated for their in vitro antimicrobial activity against both gram-positive and gram-negative bacterial strains, as well as fungal strains, using the tube dilution method. The synthesized compounds were characterized based on their physicochemical properties, and spectral data confirmed consistency with the proposed molecular structures. Docking studies, the MMGBSA analyses, and in vitro antimicrobial activity results indicated that compounds D_4_, D_6_, D_8_, and D_12_ were the most active against different microbial species and also showed favorable docking results in comparison with the PDB ligand and standard antimicrobial drugs (ofloxacin and fluconazole). This study highlights the potential of these compounds for future in vivo antimicrobial and anticancer investigations.

## 1. Introduction

The PI3K inhibitor was discovered in 1957, following its isolation from the fungal species *Penicillium wortmannii*, and was identified as a broad-spectrum compound named wortmannin [1]. Since PI3Ks are targeted by a variety of bacterial pathogens, they are considered promising targets for host-directed immune therapy, and may be beneficial managing persistent bacterial infections [2]. PI3K inhibition can improve the efficacy of treating various bacterial infections when combined with antibiotics and the host immune system [3]. The catalytic subunit of class IB phosphoinositide 3-kinases (PI3Ks), p110γ, is immune signaling and serves as a key regulator of cellular functions within class I PI3Ks [4]. In inflammatory diseases, p110γ play a vital role and has been acknowledged as a therapeutic target for inhibiting microbial synthesis and cancer cell growth due to its immunomodulatory properties [5]. Cancer and infectious diseases continue to be among the most serious health challenges faced by humanity. PI3K inhibitors have led to the development of complex biological studies and increasingly sophisticated strategies for cancer therapy. Globally, gram-negative pathogenic bacteria are becoming increasingly resistant, including strains producing extended-spectrum beta-lactamases such as *E. coli* and *Neisseria gonorrhoeae*. Based on the literature, the PI3Ks inhibitor with PDB: 7JWE was selected for the docking studies [6–7].

Schiff bases are compounds typically synthesized through the condensation reaction of amines with aldehydes or ketones [8]. They exhibit a wide range of biological actions because they may contain aromatic or substituted aliphatic side chains. These compounds are considered important intermediates in the synthesis of many different medications. The diverse biological activities of Schiff bases are the primary focus of this research, highlighting their importance as key building blocks in drug development [9]. Schiff bases demonstrate a wide array of biological effects, including antibacterial, plant growth regulator, antioxidant, enzymatic, anticancer, antiinflammatory, antimalarial, antiviral, neuroprotective, analgesic, and anticonvulsant properties, as well as neurotoxic effects [10,11]. Additionally, they are a dominating class of ligands that coordinate with metals via a variety of binding sites.

5-(pyridin-4-yl)-1,3,4-thiadiazol-2-amine cluster is a key structural component found in numerous natural products and medicinal agents, and it is considered one of the most important and well-characterized heterocycles [12,13]. 5-(pyridin-4-yl)-1,3,4-thiadiazol-2-amine nucleus is active in a variety of drug categories as a key structural aspect such as antimicrobial, antiinflammatory, palliative anticonvulsant, antifungal, antioxidant, antitubercular agents, diuretic, anticancer agents [14–20]. Extensive biochemical and pharmacological studies has shown that 5-(pyridin-4-yl)-1,3,4-thiadiazol-2-amine derivatives are beneficial toward numerous microorganisms [21–23].

## 2. Materials and methods

### 2.1. Equipment and chemicals

All starting materials used were of analytical grade and procured from various sources, including Loba Chem Pvt Ltd. and HiMedia Pvt Ltd. (New Delhi, India). Thin-layer chromatography (TLC) was performed using silica gel G as the stationary phase and a mixture of ethyl acetate and n-hexane as the mobile phase. Melting points were determined using a sonar melting point instrument (Sunbim, India). Proton nuclear magnetic resonance (^1^H NMR) spectra were recorded using a TopSpin 3.2 400 MHz NMR spectrometer (Bruker Corp., Chandigarh, India) with DMSO as the solvent. NMR compound data are reported as a wide array of the number of protons throughout the compound {singlet[s], doublet[d], triplet[t]and multiple[m]}. Infrared (IR) spectra were recorded using a Bruker 12060280 spectrophotometer (Software: OPUS 7.2.139.1294) in the range of 4000–400 cm^−1^ with KBr pellets. Mass spectra were recorded using a Micromass Q-ToF Micro instrument. A laminar airflow biosafety cabinet (Hicon, New Delhi) and a BOD incubator (Haryana Scientific and Engg. Corporation Ltd., Rohtak) were used for determining minimum inhibitory concentrations (MIC). Molecular docking studies were carried out using software developed by Schrodinger, Inc. (New York, USA). Chemical structures were drawn using ChemDraw Professional 15.0 (USA).

### 2.2. Computational studies

In silico studies, specifically molecular docking, were carried out using the Glide module of the Schrodinger Suite 2016-1 to evaluate the interaction of 1,3,4-thiadiazole derivatives as inhibitors of phosphoinositide 3-kinases (PI3Ks). Based on the literature, the crystal structure with PDB Code: 7JWE (Gedatolisib bound to the PI3Kγ catalytic subunit p110γ) was retrieved from the Protein Data Bank. The protein structure was prepared using the Protein Preparation Wizard in Schrodinger. Water molecules were removed, hydrogen atoms were added, and energy minimization was performed using the OPLS_2005 force field [24]. A grid box was created at the centroid of the protein’s active site. Grid-based ligand docking was conducted to dock the 1,3,4-thiadiazol derivatives into the catalytic domain of the protein (PDB Code: 7JWE). The Dock score, Glide Score, and MMGBSA dG parameter were used for the analysis. A total of 100 newly designed derivatives of 5-(pyridin-4-yl)-1,3,4-thiadiazol-2-amine were subjected to computational analysis, and based on the results—compared with standard drugs (ofloxacin and fluconazole)—the 17 most promising compounds were selected for synthesis [25].

### 2.3. Synthesis

**Figure 1:**
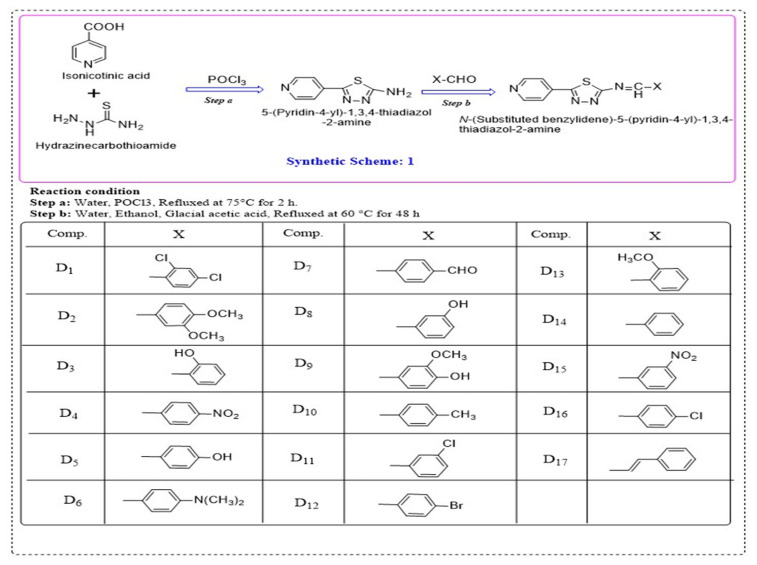
Scheme for the synthesis of Schiff’s derivatives of 1,3,4-thiadiazol-2-amine.

The 1,3,4-thiadiazole derivatives synthesized according to Scheme 1 are shown in Figure 1. General method for the synthesis of compounds D_1_–D_17_:

Step a: Synthesis of *5-(pyridin-4-yl)-1,3,4-thiadiazol-2-amine* [26]: A mixture of isonicotinic acid (50 mmol), thiosemicarbazide (50 mmol), and POCl_3_ (20 mL) was refluxed for 2 h at 75 °C. Cold water was added to the reaction mixture, and refluxing was continued for an additional 4 h. The resulting mixture was alkalized to pH 8 by the dropwise addition of 50% potassium hydroxide solution under continuous stirring. The precipitate was filtered and recrystallized using ethanol.
Step b: Synthesis of Schiff base derivatives of *5-(pyridin-4-yl)-1,3,4-thiadiazol-2-amine* [27]: A mixture of 5-(pyridin-4-yl)-1,3,4-thiadiazol-2-amine (0.27 mmol, 1.0 equiv) and a substituted aldehyde compound (1.2 equiv) was placed in a round-bottom flask and refluxed at 60 °C for 48 h in a mixture of glacial acetic acid, water and ethanol (1:1:1). The accumulated solvent was condensed off and Schiff base was obtained. The resulting precipitate was washed with water, filtered using a Whatmann filter paper, dried over anhydrous sodium sulfate, and recrystallized using methanol. The final products were purified by column chromatography using an optimized mixture of ethyl acetate and hexane as the eluent.

### 2.4. Evaluation of in vitro antimicrobial activity

The in vitro antimicrobial activity of the synthesized Schiff base derivatives of 5-(pyridin-4-yl)-1,3,4-thiadiazol-2-amine (D_1_–D_17_) was evaluated using the tube dilution method described by Cappuccino and Sherman [28], against gram-positive bacteria (*B. subtilis*, *S. aureus*), gram-negative bacteria (*E. coli*), and fungal strains (*A. niger*, *C. albicans*). The antimicrobial activity results were compared with standard drugs, namely ofloxacin (antibacterial activity) and fluconazole (antifungal activity). The tests and standard dilutions were carried out using dual strength nutrient broth I.P (for bacteria) or Sabouraud dextrose broth I.P (for fungi) as per Pharmacopoeia of India (2007) [29]. The samples were incubated for 24 h at 37 + 1 °C (bacteria), for 7 days at 25 + 1 °C (*A. niger*), and 48 h at 37 + 1 °C (*C. albicans*) [30–35].

## 3. Result and discussion

### 3.1. Computational results

Computational results were interpreted using PDB: 7JWE, which illustrates the interaction of the compounds with the PI3Ks inhibitor, as shown in Table 1.The scoring functions, including the Docking Score and Glide Score, were used to predict the binding affinities between the ligands and the target, while MM-GBSA was employed to calculate the free binding energy of the protein–ligand interactions. A stronger binding affinity is indicated by a more negative value of binding free energy. The predicted binding pattern demonstrated that derivatives interacted strongly within the catalytic cavity through π–π stacking, hydrophobic interactions, and hydrogen bonding.

**Figure 2:**
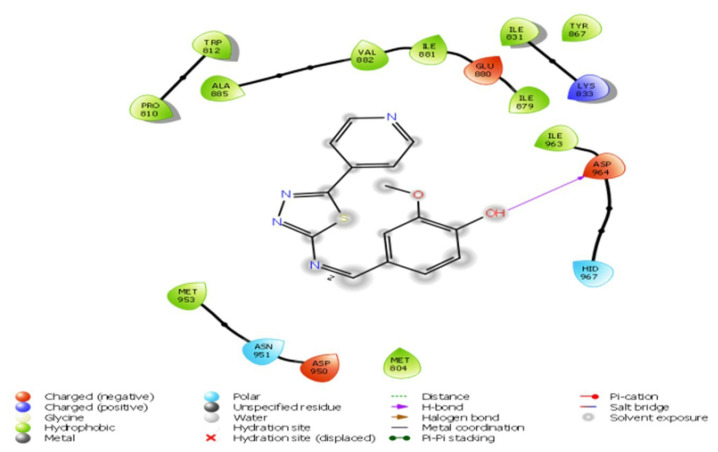
D_8_ interaction with PDB: 7JWE.

Binding analysis of compound D_8_ with PDB: 7JWE revealed that the ligand is infused into an “aromatic case” surrounded by the inhibitor-binding cavity, as demonstrated by structural pose analysis. Compound D_8_ exhibited interactions with Pro 810, Trp 512, Ala 885, Val 882, Ile 881, Glu 880, Ile 879, Ile 963, Asp 964, Met 953, Asn 951, Me 804, and Asp 950, as well as the aromatic ring of the PI3K inhibitor. Asp 964 was observed to form a hydrogen bond with an electronegative atom of the ligand, as illustrated in Figure 2.

**Figure 3:**
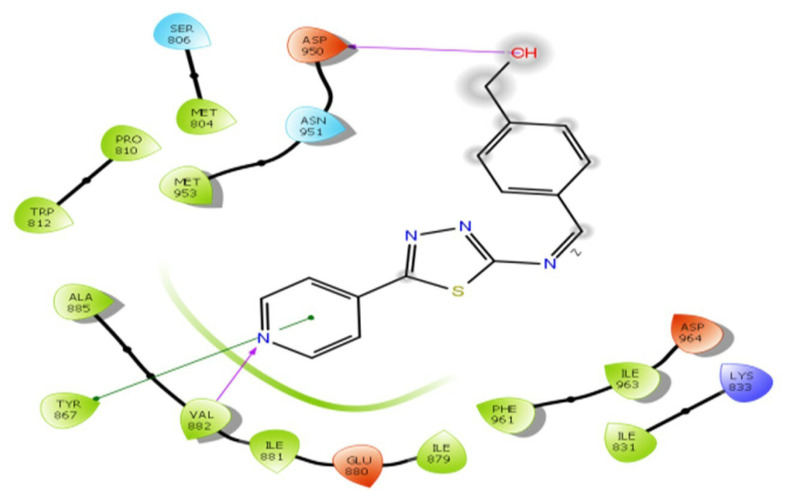
D_6_ interaction with PDB: 7JWE.

The binding results of compound D_6_ with PDB: 7JWE revealed that the ligand is infused into an “aromatic case” that is encircled by the inhibitor-binding cavity, as demonstrated by structural pose analysis. Compound D_6_ interacts with ASP 964, ILE 963, PHE 961, ILE 879, GLU 880, ILE 881, VAL 882, TYR 867, ALA 885, MET 804, SER 804, TRP 812, PRO 810, MET 953, ASN 951, and ASP 950, as well as the aromatic ring of the PI3K inhibitor. Asp 964 and Val 882 form hydrogen bonds with electronegative atoms, while Tyr 867 shows π–π stacking interaction with the N terminal of the pyridine, as shown in Figure 3.

### 3.2. Chemistry

The physicochemical properties of 5-(pyridin-4-yl)-1,3,4-thiadiazol-2-amine derivatives are presented in Table 2, and the spectral data of synthesized derivatives are as follows:

*D_1_: N-(2,4-dichlorobenzylidene)-5-(pyridin-4-yl)-1,3,4-thiadiazol-2-amine*: MS: 335.98(M^+1^); IR: 1576 (C=C str., Ar.), 2984 (C-H str., Ar.), 1681 (C=N str.,), 1047 (C-S), 826 (C-Cl); ^1^H NMR (500 MHz, DMSO-d6) δ 8.99 (s, 1H), 8.85–8.80 (m, 2H), 7.94–7.87 (m, 3H), 7.69 (d, J = 1.5 Hz, 1H), 7.47 (dd, J = 7.5, 1.5 Hz, 1H); ^13^C NMR (125 MHz, Chloroform-d) δ 175.77, 164.93, 164.92, 164.88, 162.93, 152.10, 152.09, 152.07, 152.05, 152.04, 152.03, 151.99, 138.15, 138.09, 136.68, 136.67, 136.61, 136.60, 135.71, 135.69, 132.68, 132.63, 130.68, 130.66, 130.63, 130.60, 129.48, 129.46, 129.43, 129.40, 128.71, 128.68, 128.65, 128.63, 122.29, 122.23.

*D_2_: N-(3,4-dimethoxybenzylidene)-5-(pyridin-4-yl)-1,3,4-thiadiazol-2-amine*: MS: 327.21(M^+1^); IR: 2972 (C-H str.), 1687 (C=N str.), 779(C-S str.), 1142 (C-O str.), 2894(O-CH_3_ str.), 1475 (C=C str.), 1239 (C-N str.); ^1^H NMR (500 MHz, DMSO-*d*_6_) δ 8.96 (s, 1H), 8.85–8.80 (m, 2H), 7.94–7.88 (m, 2H), 7.49–7.43 (m, 2H), 7.06 (d, *J* = 7.5 Hz, 1H), 3.83 (d, *J* = 1.0 Hz, 5H); ^13^C NMR (125 MHz, Chloroform-*d*) δ 176.49, 167.71, 167.67, 162.93, 152.41, 152.36, 152.13, 152.10, 152.09, 152.06, 152.05, 152.04, 152.03, 151.99, 150.58, 150.55, 136.68, 136.67, 136.61, 136.60, 130.07, 130.03, 125.23, 125.17, 125.15, 125.10, 122.29, 122.23, 112.51, 112.50, 112.49, 111.76, 111.74, 111.71, 111.69, 55.90, 55.88.

*D_3_: 2-((5-(pyridin-4-yl)-1,3,4-thiadiazol-2-ylimino) methyl)phenol*: MS: 283.46(M^+1^); IR: 3581 (O-H str.), 2977 (C-H str.), 1610 (C=N str.), 1480 (C=C str.), 814 (C-S str.), 1239 (C-N str.); ^1^H NMR (500 MHz, DMSO-*d*_6_) δ 8.90 (s, 1H), 8.85–8.80 (m, 2H), 7.94–7.88 (m, 2H), 7.58 (dd, *J* = 7.5, 1.5 Hz, 1H), 7.31 (td, *J* = 7.5, 1.5 Hz, 1H), 6.93 (dd, *J* = 7.5, 1.5 Hz, 1H), 6.85 (td, *J* = 7.5, 1.6 Hz, 1H); ^13^C NMR (125 MHz, Chloroform-*d*) δ 175.48, 175.42, 167.83, 167.77, 167.76, 167.72, 162.93, 160.72, 152.14, 152.13, 152.10, 152.09, 152.06, 152.05, 152.04, 152.03, 152.00, 151.99, 136.68, 136.67, 136.61, 136.60, 133.25, 133.23, 133.22, 133.19, 133.17, 133.16, 132.63, 132.58, 122.29, 122.23, 120.36, 120.34, 120.29, 119.96, 117.28, 117.27, 117.22, 117.20.

*D_4_: (E)-N-(4-nitrobenzylidene)-5-(pyridin-4-yl)-1,3,4-thiadiazol-2-amin*e: MS: 311.20; IR: 1531 (N-O str.), 818 (C-S str.), 1597 (C=N str.), 1443 (C=C str.), 2972 (C-H str.), 1233 (C-N str.); ^1^H NMR (500 MHz, DMSO-*d*_6_) δ 8.97 (s, 0H), 8.85–8.80 (m, 1H), 8.35–8.29 (m, 1H), 8.17–8.11 (m, 1H), 7.94–7.88 (m, 1H); ^13^C NMR (125 MHz, Chloroform-*d*) δ 176.47, 168.58, 168.53, 168.48, 162.93, 152.13, 152.10, 152.09, 152.07, 152.03, 152.00, 149.33, 149.32, 149.28, 149.26, 141.56, 141.52, 136.68, 136.67, 136.61, 136.60, 130.16, 130.14, 130.11, 130.09, 124.60, 124.59, 122.29, 122.23.

*D_5_: 4-((5-(pyridin-4-yl)-1,3,4-thiadiazol-2-ylimino) methyl)phenol*: MS: 283.40(M^+1^); IR: 1680 (C=N str.), 1217 (C-N str.), 829 (C-S str.), 2892 (C-H str.), 3610 (O-H str.), 1444 (C=C str.); ^1^H NMR (500 MHz, DMSO-*d*_6_) δ 8.86–8.80 (m, 3H), 8.13 (s, 1H), 7.94–7.88 (m, 2H), 7.72–7.67 (m, 2H), 6.97–6.91 (m, 2H); ^13^C NMR (125 MHz, Chloroform-*d*) δ 176.50, 176.44, 168.83, 168.78, 162.93, 156.22, 152.13, 152.10, 152.09, 152.07, 152.03, 152.00, 136.67, 136.60, 131.88, 131.86, 131.83, 131.82, 131.81, 127.66, 127.60, 122.29, 122.23, 116.58, 116.55, 116.53, 116.50.

*D_6_: N-(4-(dimethylamino)benzylidene)-5-(pyridin-4-yl)-1,3,4-thiadiazol-2-amine*: MS: 310.11(M^+1^); IR: 1592 (C=N str.), 2913 (N-CH_3_ str.), 1440 (C=C str.), 813 (C-S str.), 1232 (C-N str.); ^1^H NMR (500 MHz, DMSO-*d*_6_) δ 8.85–8.80 (m, 1H), 7.94–7.88 (m, 1H), 7.82–7.76 (m, 1H), 6.74–6.69 (m, 1H), 3.00 (s, 2H); ^13^C NMR (125 MHz, Chloroform-*d*) δ 176.47, 168.88, 168.83, 162.93, 152.10, 152.09, 152.06, 152.04, 152.03, 152.00, 151.99, 151.94, 151.93, 136.68, 136.67, 136.60, 131.10, 131.08, 131.07, 131.05, 131.02, 125.90, 125.86, 122.29, 122.23, 111.60, 111.59, 111.58, 40.27.

*D_7_: 4-((5-(pyridin-4-yl)-1,3,4-thiadiazol-2-ylimino)methyl) benzaldehyde*: MS: 295.11(M^+1^); IR: 1628 (C=N str.), 809 (C-S str.), 1244 (C-N str.), 1483 (C=C str.), 2978 (C-H str.), 1683(C=O str.); ^1^H NMR (500 MHz, DMSO-*d*_6_) δ 9.95 (s, 1H), 8.86–8.80 (m, 3H), 8.00–7.94 (m, 2H), 7.94–7.88 (m, 2H), 7.85–7.79 (m, 2H); ^13^C NMR (125 MHz, Chloroform-*d*) δ 192.34, 192.18, 192.15, 192.11, 176.50, 176.44, 168.94, 168.89, 162.93, 152.13, 152.10, 152.09, 152.06, 152.03, 152.00, 141.42, 141.40, 141.36, 141.35, 137.61, 137.42, 136.68, 136.67, 136.61, 136.60, 130.01, 129.98, 129.96, 129.92, 129.91, 129.87, 129.86, 122.29, 122.23.

*D_8_: (E)-3-((5-(pyridin-4-yl)-1,3,4-thiadiazol-2-ylimino)methyl)phenol*: MS: 283.52(M^+1^); IR: 3737 (O-H str.), 2925 (C-H str.), 1600 (C=N str.), 1451(C=C str.), 840 (C-S str.), 1226 (C-N str.); ^1^H NMR (500 MHz, DMSO-*d*_6_) δ 8.85–8.78 (m, 3H), 7.94–7.88 (m, 2H), 7.79 (s, 1H), 7.51 (dt, *J* = 7.8, 1.7 Hz, 1H), 7.30 (t, *J* = 1.5 Hz, 1H), 7.20 (t, *J* = 7.5 Hz, 1H), 7.11 (dt, *J* = 7.5, 1.5 Hz, 1H); ^13^C NMR (125 MHz, Chloroform-*d*) δ 176.51, 176.45, 167.65, 167.60, 167.55, 162.93, 158.30, 152.14, 152.13, 152.10, 152.09, 152.07, 152.06, 152.05, 152.04, 152.03, 152.00, 151.99, 137.75, 137.73, 136.68, 136.67, 136.61, 136.60, 130.01, 130.00, 129.99, 129.98, 122.68, 122.66, 122.63, 122.61, 122.29, 122.23, 115.65, 115.63, 115.59, 115.58, 115.54, 115.52, 114.99, 114.97, 114.93, 114.92.

*D_9_: 2-methoxy-4-((5-(pyridin-4-yl)-1,3,4-thiadiazol-2-ylimino)methyl) phenol*: MS: 313.16(M^+1^); IR: 3664 (O-H str.), 2885 (O-CH_3_ str.), 810 (C-S str.), 1265 (C-N str.), 3153 (C-H str.), 1428 (C=C str.), 1662(C=N str.); ^1^H NMR (500 MHz, DMSO-*d*_6_) δ 8.87 (s, 1H), 8.85–8.80 (m, 2H), 8.66 (s, 1H), 7.94–7.88 (m, 2H), 7.35 (d, *J* = 1.6 Hz, 1H), 7.14 (dd, *J* = 7.6, 1.5 Hz, 1H), 6.89 (d, *J* = 7.5 Hz, 1H), 3.87 (s, 3H); ^13^C NMR (125 MHz, Chloroform-*d*) δ 176.51, 176.45, 167.91, 167.90, 167.85, 162.93, 152.14, 152.13, 152.10, 152.09, 152.06, 152.05, 152.04, 152.03, 152.00, 151.99, 148.14, 147.27, 136.68, 136.67, 136.61, 136.60, 127.67, 127.65, 125.30, 125.28, 125.24, 125.23, 125.19, 125.17, 122.29, 122.23, 114.99, 114.98, 111.92, 111.90, 56.21.

*D_10_: N-(4-methylbenzylidene)-5-(pyridin-4-yl)-1,3,4-thiadiazol-2-amine*: MS: 281.08(M^+1^); IR: 2909 (C-CH_3_ str.), 2975 (C-H str.), 1596 (C=N str.), 893 (C-S str.), 1376 (C-N str.), 1479 (C=C str.); ^1^H NMR (500 MHz, DMSO-*d*_6_) δ 8.97 (s, 1H), 8.85–8.80 (m, 2H), 7.94–7.88 (m, 2H), 7.84–7.78 (m, 2H), 7.20 (dq, *J* = 7.1, 0.8 Hz, 2H), 2.40 (d, *J* = 0.9 Hz, 3H); ^13^C NMR (125 MHz, Chloroform-*d*) δ 176.47, 168.76, 168.71, 162.93, 152.13, 152.10, 152.09, 152.07, 152.03, 152.00, 140.57, 140.56, 140.50, 136.67, 136.60, 133.08, 133.04, 129.79, 129.77, 129.74, 129.72, 129.01, 128.99, 128.98, 128.97, 128.96, 128.95, 122.29, 122.23, 21.40, 21.37, 21.33.

*D_11_: N-(3-chlorobenzylidene)-5-(pyridin-4-yl)-1,3,4-thiadiazol-2-amine*: MS: 301.15; IR: 2818 (C-H str.), 1271 (C-N str.), 1530 (C=C str.), 834 (C-S str.), 1696 (C=N str.), 743 (C-Cl str.); ^1^H NMR (500 MHz, DMSO-*d*_6_) δ 8.97 (s, 1H), 8.85–8.80 (m, 2H), 7.96–7.88 (m, 3H), 7.81 (dt, *J* = 6.9, 1.8 Hz, 1H), 7.59–7.50 (m, 2H); ^13^C NMR (125 MHz, Chloroform-*d*) δ 176.48, 167.99, 167.95, 167.91, 167.90, 167.86, 162.93, 152.14, 152.13, 152.10, 152.09, 152.07, 152.06, 152.05, 152.04, 152.03, 152.00, 151.99, 137.16, 137.12, 136.68, 136.67, 136.61, 136.60, 133.63, 133.62, 133.60, 133.60, 131.74, 131.73, 131.69, 131.67, 131.63, 131.62, 129.62, 129.60, 129.56, 129.54, 128.41, 128.40, 128.39, 127.56, 127.55, 127.51, 127.50, 122.29, 122.23.

*D_12_: N-(4-bromobenzylidene)-5-(pyridin-4-yl)-1,3,4-thiadiazol-2-amine*: MS: 345.16(M^+1^); IR: 2952 (C-H str.), 1573 (C=C str.), 1270 (C-N str.), 847 (C-S str.), 1573 (C=N str.), 664 (C-Br str.); ^1^H NMR (500 MHz, DMSO-*d*_6_) δ 8.97 (s, 0H), 8.85–8.80 (m, 1H), 7.94–7.88 (m, 1H), 7.86–7.80 (m, 1H), 7.74–7.68 (m, 1H); ^13^C NMR (125 MHz, Chloroform-*d*) δ 176.47, 168.71, 168.66, 162.93, 152.13, 152.10, 152.09, 152.07, 152.03, 152.00, 136.68, 136.67, 136.61, 136.60, 135.06, 135.01, 131.71, 131.70, 131.69, 130.59, 130.56, 130.53, 130.51, 124.57, 124.56, 124.52, 124.50, 122.29, 122.23.

*D_13_: N-(2-methoxybenzylidene)-5-(pyridin-4-yl)-1,3,4-thiadiazol-2-amine*: MS: 297.13(M^+1^); IR: 3074 (C-H str.), 1351 (C-N str.), 823 (C-S str.), 1697 (C=N str.), 1441 (C=C str.), 2819 (O-CH_3_ str.); ^1^H NMR (500 MHz, DMSO-*d*_6_) δ 8.95 (s, 1H), 8.85–8.80 (m, 2H), 7.94–7.88 (m, 2H), 7.78 (dd, *J* = 7.7, 1.6 Hz, 1H), 7.43 (td, *J* = 7.4, 1.5 Hz, 1H), 7.11 (dd, *J* = 7.5, 1.6 Hz, 1H), 7.04 (td, *J* = 7.5, 1.5 Hz, 1H), 3.83 (s, 2H); ^13^C NMR (125 MHz, Chloroform-*d*) δ 175.58, 166.43, 166.38, 166.33, 166.29, 162.93, 161.01, 160.96, 152.14, 152.13, 152.10, 152.09, 152.06, 152.05, 152.04, 152.03, 151.99, 136.68, 136.67, 136.61, 136.60, 131.30, 131.25, 131.24, 131.22, 131.17, 131.16, 131.11, 131.10, 128.62, 128.58, 122.29, 122.23, 121.10, 121.06, 121.04, 121.02, 113.85, 113.84, 113.83, 113.79, 113.78, 113.77, 55.79.

*D_14_: N-benzylidene-5-(pyridin-4-yl)-1,3,4-thiadiazol-2-amine*: MS: 267.18(M^+1^); IR: 1697 (C=N str.), 716 (C-S str.), 1273 (C-H str.), 3040 (C-H str.), 1532 (C=C str.); ^1^H NMR (500 MHz, DMSO-*d*_6_) δ 8.92 (s, 1H), 8.85–8.80 (m, 2H), 7.94–7.86 (m, 4H), 7.39–7.32 (m, 1H), 7.21–7.14 (m, 2H); ^13^C NMR (125 MHz, Chloroform-*d*) δ 176.47, 168.73, 168.68, 162.93, 152.13, 152.10, 152.09, 152.07, 152.03, 152.00, 136.68, 136.67, 136.61, 136.60, 136.07, 136.03, 130.86, 130.85, 130.80, 129.79, 129.77, 129.73, 129.72, 129.67, 129.66, 128.85, 128.84, 128.83, 128.82, 122.29, 122.23.

*D_15_: N-(3-nitrobenzylidene)-5-(pyridin-4-yl)-1,3,4-thiadiazol-2-amine*: MS: 312.21(M^+1^); IR: 1533 (N-O str.), 1646 (C=N str.), 2978 (C-H str.), 810 (C-S str.), 1449 (C=C str.), 1241 (C-N str.); ^1^H NMR (500 MHz, DMSO-*d*_6_) δ 9.13 (s, 1H), 8.85–8.80 (m, 2H), 8.55–8.51 (m, 1H), 8.15 (dt, *J* = 7.5, 1.6 Hz, 1H), 8.04 (dt, *J* = 7.5, 1.6 Hz, 1H), 7.94–7.88 (m, 2H), 7.62 (t, *J* = 7.5 Hz, 1H); ^13^C NMR (125 MHz, Chloroform-*d*) δ 176.48, 168.66, 168.61, 168.57, 168.52, 162.93, 152.14, 152.13, 152.10, 152.09, 152.07, 152.06, 152.05, 152.04, 152.03, 152.00, 151.99, 145.86, 145.84, 137.06, 137.01, 136.68, 136.67, 136.61, 136.60, 133.56, 133.54, 133.51, 133.49, 129.30, 129.28, 129.28, 129.27, 124.27, 124.26, 124.22, 124.21, 122.82, 122.80, 122.76, 122.75, 122.71, 122.69, 122.29, 122.23.

*D_16_: N-(4-Chlorobenzylidene)-5-(pyridin-4-yl)-1,3,4-thiadiazol-2-amine*: MS: 301.15(M^+1^); IR: 1604 (C=N str.), 851 (C-Cl str.), 815 (C-S str.), 1291 (C-N str.), 2847 (C-H str.), 3391 (N-H str.); ^1^H NMR (500 MHz, DMSO-*d*_6_) δ 8.97 (s, 0H), 8.85–8.80 (m, 1H), 7.94–7.88 (m, 1H), 7.83–7.77 (m, 1H), 7.72–7.66 (m, 1H); ^13^C NMR (125 MHz, Chloroform-*d*) δ 176.47, 168.60, 168.55, 162.93, 152.13, 152.10, 152.09, 152.07, 152.03, 152.00, 138.26, 138.23, 138.19, 138.16, 136.67, 136.60, 134.75, 134.70, 131.08, 131.05, 131.03, 131.00, 130.98, 129.43, 129.42, 129.40, 129.39, 122.29, 122.23.

*D_17_: (E)-N-(3-Phenylallylidene)-5-(pyridin-4-yl)-1,3,4-thiadiazol-2-amine*: MS: 293.08(M^+1^); IR: 1678 (C=N str.), 1615 (C=C str.), 870 (C-S str.), 2841 (C-H str.), 1253 (C-N str.); ^1^H NMR (500 MHz, DMSO-*d*_6_) δ 8.85–8.80 (m, 2H), 7.97 (dd, *J* = 6.0, 0.7 Hz, 1H), 7.94–7.88 (m, 2H), 7.81–7.75 (m, 2H), 7.43–7.36 (m, 2H), 7.35–7.28 (m, 1H), 6.88 (dd, *J* = 15.0, 6.0 Hz, 1H), 6.77 (dd, *J* = 15.1, 0.8 Hz, 1H); ^13^C NMR (125 MHz, Chloroform-*d*) δ 174.91, 162.83, 159.51, 159.50, 159.45, 159.44, 159.39, 159.38, 152.13, 152.10, 152.09, 152.06, 152.03, 152.00, 143.44, 143.43, 143.41, 143.38, 143.37, 143.34, 136.68, 136.67, 136.61, 136.60, 136.19, 136.13, 129.46, 129.42, 129.40, 129.39, 129.34, 128.82, 128.81, 128.79, 128.78, 128.77, 127.75, 127.74, 127.72, 127.69, 127.67, 127.64, 127.62, 122.29, 122.23.

### 3.3. Antimicrobial screening results

All the newly synthesized compounds of 5-(pyridin-4-yl)-1,3,4-thiadiazol-2-amine derivatives were investigated for their in vitro antimicrobial potential against different microbial strains, such as gram-positive and gram-negative bacteria, as well as fungi, using the tube dilution technique. The results are presented in Table 3. The results of the antimicrobial study revealed that the synthesized compounds displayed significant antimicrobial activity against the tested species of microorganisms. Compounds D_4_, D_8_, and D_11_ (pMIC= 1.9;1.9;1.9 μM/mL) were found to be particularly effective against *E. coli.* Compounds D_4_, D_8_, and D_11_ (pMIC = 1.9; 1.9; 1.9 μM/mL) were found to be the most effective against *B. subtilis*, and compounds D_12_, D_7_, and D_4_ (pMIC = 2.04; 2.16; 2.28 μM/mL) were also found to be effective against *S. aureus.* Antifungal activity results indicated that compounds D_4_ and D_8_ (pMIC = 2.2; 1.95 μM/mL) were primarily active against *C*. *albicans*, and compounds D_5_, D_6_, and D_8_ (pMIC = 1.95; 1.95; 1.99 μM/mL) were most active against *A*. *niger*. Among the synthesized compounds, molecule D_4_ exhibited tremendous antibacterial potential, while molecule D_8_ demonstrated remarkable antifungal activity. Both could serve as lead compounds for the future development of novel antibacterial and antifungal medicines.

### 3.4. Structure–activity relationship (SAR)

**Figure 4:**
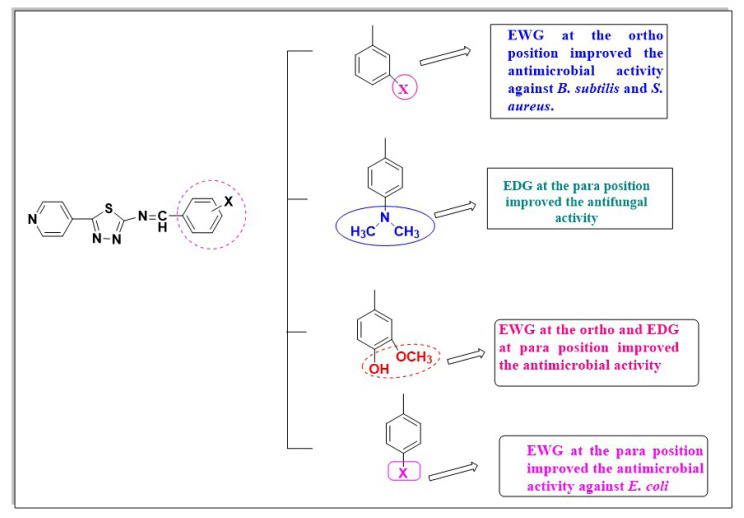
Structure activity relationship of the synthesized compounds.

❖ The following structure–activity relationship (SAR) can be derived from the antimicrobial screening scores of the synthesized 5-(pyridin-4-yl)-1,3,4-thiadiazol-2-amine derivatives (Figure 4):
❖ The presence of electron-withdrawing group at the meta position (Cl, Br, Nitro) in compounds D_11_ and D_15_ enhanced antimicrobial activity against *B. subtilis* and *S*. aureus.
❖ The presence of electron-withdrawing group at the para position (Cl, Br, Nitro) in derivatives D_4_, D_7_, D_12_, and D_16_ enhanced the antimicrobial activity against *E. coli*.
❖ The presence of electron-donating group at the para position (i.e. dimethylamino group [-N(CH_3_)_2_]) in derivatives D_6_, D_5_, and D_10_ improved antifungal activity against *C. albicans.* The presence of (–OH) and (–OCH_3_) at the ortho and para positions, respectively, increased antifungal activity against *A*. *niger.*
❖ The presence of electron-withdrawing group at the para and ortho positions in derivatives D_1_ decreased antimicrobial activity.

## 4. Conclusion

In the present study, a series of 5-(pyridin-4-yl)-1,3,4-thiadiazol-2-amine derivatives were designed and tested through docking studies against *PI3Ks inhibitor* PDB: 7JWE. Based on the docking results, compounds D_1_ and D_17_ were synthesized in significant yields and evaluated for their in vitro antimicrobial activity using the tube dilution method. The synthesized compounds were characterized by their physicochemical properties, and spectral data confirmed their alignment with the designed molecular structures. The docking and in vitro antimicrobial activity results showed a strong correlation. Some of the derivatives showed adequate activity against different microbial strains, with superior docking scores compared to standard drugs. The in vitro antimicorbial results suggest that these compounds are promising candidates for in vivo evaluation. The docking results also revealed the potential of these compounds for PI3K inhibition, indicating their possible application in breast cancer treatment through a multifaceted network of sophisticated strategies [36].

## Figures and Tables

**Table 1:** Docking results of the synthesized compounds against PDB: 7JWE.

Title	Docking Score	Glide Score	MMGBSA dG bind (NS)
**Ligand structure gedatolisib**	−10.607	−10.607	−140.66
**D** **_1_** **.mol**	−5.695	−5.695	−66.35
**D** **_2_** **.mol**	−7.159	−7.159	−64.67
**D** **_3_** **.mol**	−6.078	−6.078	−66.46
**D** **_4_** **.mol**	−6.817	−6.817	−67.07
**D** **_5_** **.mol**	−6.461	−6.461	−71.89
**D** **_6_** **.mol**	−7.251	−7.251	−69.18
**D** **_7_** **.mol**	−7.073	−7.073	−66.64
**D** **_8_** **.mol**	−7.617	−7.617	−66.55
**D** **_9_** **.mol**	−6.087	−6.087	−66.74
**D** **_10_** **.mol**	−6.201	−6.201	−65.02
**D** **_11_** **.mol**	−6.267	−6.267	−61.47
**D** **_12_** **.mol**	−5.731	−5.731	−63.81
**D** **_13_** **.mol**	−6.291	−6.291	−65.31
**D** **_14_** **.mol**	−6.484	−6.484	−64.9
**D** **_15_** **.mol**	−6.034	−6.034	−65.22
**D** **_16_** **.mol**	−5.911	−5.911	−65.22
**D** **_17_** **.mol**	−5.498	−5.498	−65.87
**Ofloxacin.mol**	−7.179	−7.179	−67.83
**Fluconazole.mol**	−5.116	−5.116	−43.1

**Table 2:** Physicochemical properties of the synthesized compounds.

Compound	Molecular formula	Molecular weight	Melting point (°C)	R*_f_* value	% yield
**D** **_1_**	C_14_H_8_Cl_2_N_4_S	335.21	282–285	0.33	36
**D** **_2_**	C_16_H_14_N_4_O_2_S	326.08	230–233	0.61	48
**D** **_3_**	C_14_H_10_N_4_OS	282.32	229–232	0.67	48
**D** **_4_**	C_14_H_9_N_5_O_2_S	311.05	261–264	0.31	46
**D** **_5_**	C_14_H_10_N_4_OS	282.32	221–224	0.72	41
**D** **_6_**	C_16_H_15_N_5_S	309.39	236–239	0.57	48
**D** **_7_**	C_22_H_14_N_8_S_2_	454.53	231–234	0.51	39
**D** **_8_**	C_14_H_10_N_4_OS	282.32	228–231	0.68	34
**D** **_9_**	C_15_H_12_N_4_O_2_S	312.35	236–239	0.54	49
**D** **_10_**	C_15_H_12_N_4_S	280.35	214–217	0.76	42
**D** **_11_**	C_14_H_9_ClN_4_S	300.77	242–245	0.45	37
**D** **_12_**	C_14_H_9_BrN_4_S	345.22	247–249	0.41	35
**D** **_13_**	C_15_H_12_N_4_OS	296.35	224–227	0.64	44
**D** **_14_**	C_14_H_10_N_4_S	266.32	213–216	0.82	37
**D** **_15_**	C_14_H_9_N_5_O_2_S	311.32	248–251	0.36	43
**D** **_16_**	C_14_H_9_ClN_4_S	300.77	235–237	0.48	34
**D** **_17_**	C_16_H_12_N_4_S	292.36	217–220	0.79	36

* TLC mobile phase: ethylacetate: n-hexane (5:5).

**Table 3:** Antimicrobial screening results of the synthesized compounds

Comp. No.	pMIC values in μM/mL				
	Bacterial species			Fungal species	
	*E. coli*	*B. subtilis*	*S. aureus*	*C. albicans*	*A. niger*
**D** **_1_**	1.428406953	1.428406953	1.428406953	1.127376957	1.127376957
**D** **_2_**	1.717444145	1.717444145	1.717444145	1.416414149	1.115384154
**D** **_3_**	1.353831632	1.353831632	1.353831632	1.353831632	1.052801637
**D** **_4_**	1.980946845	1.980946845	2.299705607	1.395920193	1.395920193
**D** **_5_**	1.654861628	1.654861628	1.654861628	1.956587051	1.956587051
**D** **_6_**	1.694626255	1.694626255	1.694626255	2.297381674	1.995656251
**D** **_7_**	1.861682536	1.861682536	2.162712531	1.56065254	1.56065254
**D** **_8_**	1.955891624	1.955891624	1.654861628	1.955891624	1.955891624
**D** **_9_**	1.397731497	1.397731497	1.397731497	1.096701502	1.096701502
**D** **_10_**	1.350790547	1.350790547	1.350790547	1.651820543	1.350790547
**D** **_11_**	1.984079922	1.984079922	1.682354498	1.381324503	1.080294507
**D** **_12_**	1.742215931	1.742215931	2.043245927	1.441185935	1.14015594
**D** **_13_**	1.675924914	1.675924914	1.374894919	1.675924914	1.675924914
**D** **_14_**	1.328493769	1.328493769	1.328493769	1.328493769	1.328493769
**D** **_15_**	1.999052428	1.999052428	1.697327004	1.396297009	1.095267013
**D** **_16_**	1.381324503	1.381324503	1.682354498	1.080294507	0.779264511
**D** **_17_**	1.36900794	1.36900794	1.36900794	1.067977945	0.766947949
**Std.(a)**	**1.762076**	**1.762076**	**1.762076**	**-**	**-**
**Std.(b)**	-	-	-	**1.690224441**	**1.389194446**

Std. aOfloxacin; bFluconazole.
